# Variation of mutant allele frequency in *NRAS* Q61 mutated melanomas

**DOI:** 10.1186/s12895-017-0061-x

**Published:** 2017-07-01

**Authors:** Zofia Hélias-Rodzewicz, Elisa Funck-Brentano, Nathalie Terrones, Alain Beauchet, Ute Zimmermann, Cristi Marin, Philippe Saiag, Jean-François Emile

**Affiliations:** 1 Research Unit EA4340 Biomarkers in Cancerology and Hemato Oncology, Versailles SQY University, Paris-Saclay University, 9, Avenue Charles de Gaulle, 92104 Boulogne-Billancourt, France; 2 Department of Pathology, Ambroise Paré Hospital, AP-HP, Boulogne-Billancourt, France; 3 Department of Dermatology, Ambroise Paré Hospital, AP-HP, Boulogne-Billancourt, France; 4 Department of Public Health, Ambroise Paré Hospital Ap-HP, Boulogne-Billancourt, France

**Keywords:** Melanoma, M%*NRAS*, Imbalance, Pyrosequencing, WT allele loss

## Abstract

**Background:**

Somatic mutations of *BRAF* or *NRAS* activating the MAP kinase cell signaling pathway are present in 70% of cutaneous melanomas. The mutant allele frequency of *BRAF* V600E (M%*BRAF*) was recently shown to be highly heterogeneous in melanomas. The present study focuses on the *NRAS* Q61 mutant allele frequency (M%*NRAS*).

**Methods:**

Retrospective quantitative analyze of 104 *NRAS* mutated melanomas was performed using pyrosequencing. Mechanisms of M%*NRAS* imbalance were studied by fluorescence in situ hybridization (FISH) and microsatellite analysis.

**Results:**

M%*NRAS* was increased in 27.9% of cases. FISH revealed that chromosome 1 instability was the predominant mechanism of M%*NRAS* increase, with chromosome 1 polysomy observed in 28.6% of cases and intra-tumor cellular heterogeneity with copy number variations of chromosome 1/*NRAS* in 23.8%. Acquired copy-neutral loss of heterozygosity (LOH) was less frequent (19%). However, most samples with high M%*NRAS* had only one copy of *NRAS* locus surrounding regions suggesting a WT allele loss. Clinical characteristics and survival of patients with either <60% or ≥60% of M%*NRAS* were not different.

**Conclusion:**

As recently shown for M%*BRAF*, M%*NRAS* is highly heterogeneous. The clinical impacts of high M%*NRAS* should be investigated in a larger series of patients.

**Electronic supplementary material:**

The online version of this article (doi:10.1186/s12895-017-0061-x) contains supplementary material, which is available to authorized users.

## Background

Cutaneous melanoma is a highly aggressive and treatment-resistant human cancer. The most frequent genetic alterations involve genes of the MAP kinase signaling pathway [1–3]. Activating hot-spot mutations are mainly found in *BRAF* (codon V600) and in *NRAS* (codon Q61, and less frequently in the codons G12 and G13) genes, in 35–50% and 15–25% of cutaneous melanoma, respectively [4, 5]. Among *BRAF* alterations, the *BRAF* V600E mutation in exon 15 is predominant (85%) and due to a substitution of a valine to a glutamic acid (c.1799 T > A, p.V600E) [6, 7]. *BRAF* and *NRAS* mutations are almost always mutually exclusive [8, 9].

Mutant *NRAS* melanomas have been reported to have more aggressive clinical features than other subtypes, with thicker lesions, elevated mitotic activity, and higher rates of lymph node metastasis [10–12]. Additionally, *NRAS* mutation status was reported as a predictor of poorer outcomes with lower median survival compared to non-*NRAS* mutated melanoma [10, 13].

The discovery of *BRAF* mutations led to the development of targeted treatments [14, 15]. However despite major clinical benefit in melanomas with *BRAF* mutation, secondary resistance occurs in most patients during the first year of treatment. Thus combinations of BRAF and MEK inhibitors have been developed, and were shown to induce longer progression free survivals (PFS) of patients with *BRAF* mutated melanomas [16–18]. By contrast, targeted treatment of patients with *NRAS* mutated melanomas is still a challenge, although an international phase 3 prospective study with the MEK inhibitor binimetinib recently provided promising results [19].

We recently studied the frequency of *BRAF* mutant alleles (M%*BRAF)* and showed that M%*BRAF* is highly heterogeneous and frequently increased in *BRAF* mutated melanomas [20]. Interestingly, a recent clinical study showed that the increased *BRAF* V600 mutation level was significantly associated with a better response rate to vemurafenib during the first 10 months of treatment [21]. These observations highlighted the importance of quantitative evaluation of *BRAF* mutation before melanoma treatment.

Although biological and clinical implication of the frequency of mutant alleles of *BRAF* in melanomas are currently under investigation, no data are available concerning the variation of M%*NRAS*. Accordingly, we conducted this study to investigate *NRAS* Q61 mutations and M%*NRAS* in a series of 199 melanomas wild type for *BRAF* V600. The mechanisms of the M%*NRAS* variations were then studied by fluorescence in situ hybridization (FISH) and by amplified fragment length polymorphism (AFLP).

## Methods

### Patients and samples

Melanoma samples were obtained from the bank of biological resources of Ambroise Paré Hospital in Boulogne-Billancourt. The research was performed in compliance with the ethical principles of the Helsinki Declaration (1964) and with the French ethics laws. Patients were informed and approved the use of their samples for research purpose. Tumor samples collection was declared to the French Ministry of Research (DC 2009–933). Melan-Cohort study was approved by CPP IDF 8 Ethics committee (030209) and registered with https://www.clinicaltrials.gov/ct2/search (NCT00839410). Clinical and survival data were collected from clinical records of the Dermatology Department of Ambroise Paré Hospital.

The frequency of *NRAS* Q61 mutations was evaluated in a consecutive series of melanomas received for diagnosis from March 2013 until May 2015. Additionally, a second series of patients, whose samples were received earlier to March 2013, mutated for *NRAS* were also included for the evaluation of M%*NRAS*. In our previous paper concerning the *BRAF* mutant allele frequency in melanoma [20], we observed a distinct distribution of the percentage of mutated allele according to the percentage of tumor cells. However, the inter-pathologist reproducibility for the evaluation of tumor cell content was substantial for the 80% cut-off (κ = 0.79). Therefore, we excluded samples with less than 80% of melanoma cells from further *NRAS* molecular analysis.

### *NRAS* mutant allele detection and quantification

Before DNA extraction, HES slides were reviewed to confirm the presence of melanoma cells and to select areas with highest density of tumor cells for macrodissection. For microsatellite analysis, DNA from corresponding normal tissue section was extracted. Genomic DNA was extracted from formalin-fixed and paraffin-embedded (FFPE) fragments of melanoma as previously described [22].

Pyrosequencing was performed as already described [23]. Profiles for different *NRAS* mutations were established and confirmed by Sanger sequencing method (Additional file 1: Figure S1). Three different assays for detection of *NRAS* Q61 mutation were designed and primers used for DNA amplification, pyrosequencing, and Sanger method are presented in Table 1. Pyrosequencing Assay 1 allows the quantification of all but one *NRAS* Q61 mutation (c.183A > T p.Q61H) [24]. Pyrosequencing Assay 2 is an edited version of Assay 1, in which the order of injected nucleotides was modified to allow the quantification of all *NRAS* Q61 mutations. Pyrosequencing Assay 3 was developed to rescue some cases with a very bad FFPE DNA quality. For patients with several samples available, the M%*NRAS* used was that obtained from the first metastasis.

**Table 1:** Primers and pyrosequencing assay information. Primers sequences used for PCR, Sanger sequencing, pyrosequencing and AFLP technique

NRAS Pyrosequencing ASSAY 1	
*PCR 124 nt*	
Primer F-Biotine	ACACCCCCAGGATTCTTACAGA
Primer R	GCCTGTCCTCATGTATTGGTC
Pyrosequencing primer	CATGGCACTGTACTCTTC
Nucleotide injection order	GTTACGTCAGCTG
NRAS Pyrosequencing ASSAY 2	
*PCR 124 nt*	
Primer F-Biotine	ACACCCCCAGGATTCTTACAGA
Primer R	GCCTGTCCTCATGTATTGGTC
Pyrosequencing primer	CATGGCACTGTACTCTTC
Nucleotide injection order	GCATACGTCAG
NRAS Pyrosequencing ASSAY 3	
*PCR 90 nt*	
Primer F-Biotine	ACAAGTGGTTATAGATGGTGA
Primer R	ATGTATTGGTCTCTCATGGCA
Pyrosequencing primer	CATGGCACTGTACTCTTC
Nucleotide injection order	GCATACGTCAGCT
Sanger Sequencing	
Primer F	ACAAGTGGTTATAGATGGTGA
Primer R	ATGTATTGGTCTCTCATGGCA
AFLP primers	
rs3219599 F	TTCAAGGCTGCAGTGAGCTA
rs3219599 R	AGTGGAAGCTAGACACACATTAAGA
s3219653 F	CCAGAGAGACAGAACTGAACAAA
s3219653 R	CAAATTTTGGACCTGCCATG
rs3219587 F	GGGCAAATGGAGGAAAGAGA
rs3219587 R	TAAAAATACCCCCACCCCACT
rs3220698 F	TTAAAAAACGTACTGCCACATTCA
rs3220698 R	GGCAGAAACCAGGAAATGTAGTA
rs3220987 F	GGCTTTTAGCTATGATTTGAGA
rs3220987 R	GACTCAGGAAATAAACAAGGC
rs3220389 F	CGCTGCTCACTCCTCCTCTGA
rs3220389 R	AGTGCTGCTCTCAGTGAACTC
rs3219612 F	AGCACACAATATACTCTCTCAGA
rs3219612 R	ACCTGGGCAAAAGAGTAAGACC
rs3219703 F	AACGAAGGTGTACTGGGACTGGT
rs3219703 R	ACAGGGATGTGAGGGATTTTTTC

### Microsatellite analysis

**Fig. 1:**
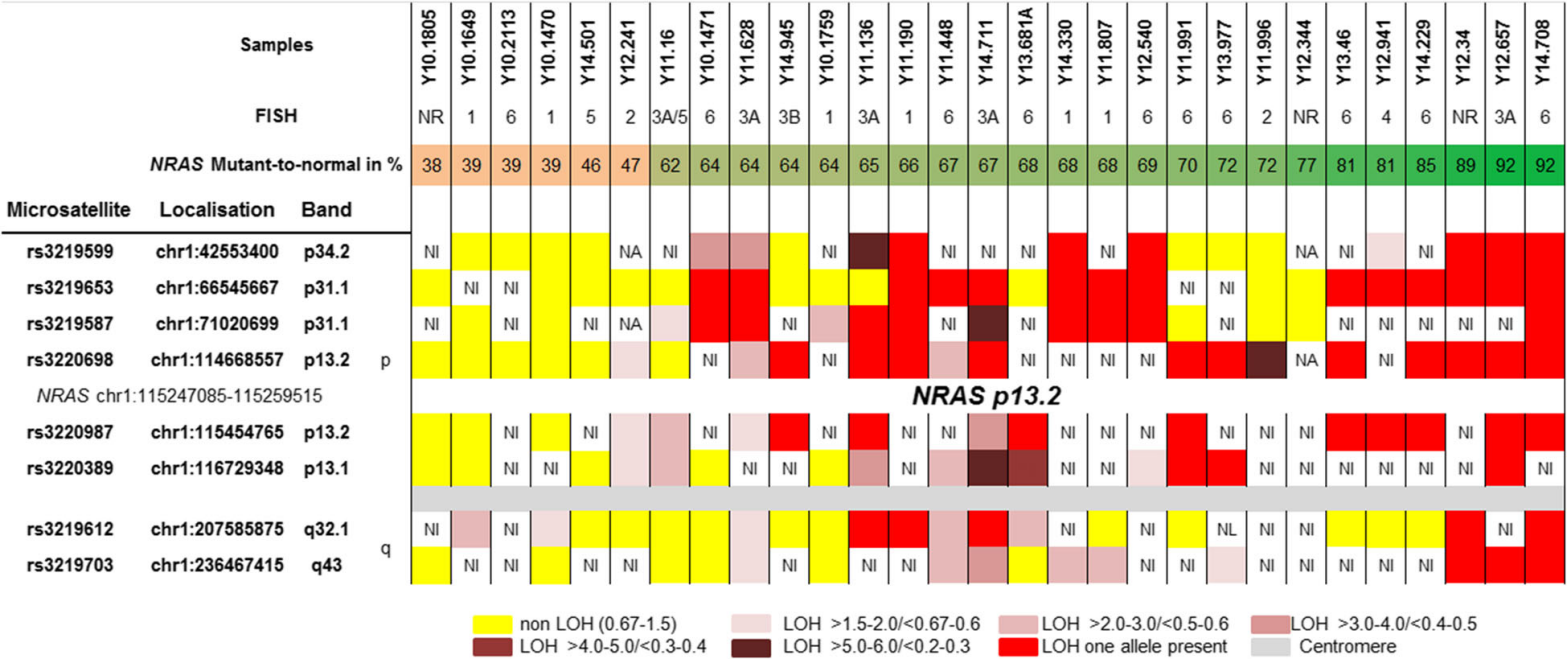
Chromosome 1 microsatellite analyses in *NRAS* mutated melanomas. Summary of AFLP analysis of eight chromosome 1 microsatellite markers performed in 29 *NRAS* Q61 mutated melanomas. Each row contains following information for one patient: identification n°, *NRAS*/chromosome 1 FISH result, *NRAS* mutant allele percentage, results of microsatellite markers LOH. *NRAS* gene and centromere localization are indicated. NA – non analysable, NI – non informative, NR – not realized. FISH: 1 - disomy, 2 - disomy with rares polysomic cells, 3 polysomy (A:3–4 copy, B: >4 copy), 4 - amplification, 5- monosomy, 6- high intra-tumor copy number variations of *NRAS*/chromosome 1

Eight microsatellite markers were selected from the NCBI dbSNP short genetic variations database and analyzed by AFLP. Their positions are showed in Fig. 1, and the primers used in Table 1. No highly heterozygous microsatellite was identified within *NRAS* gene; thus the genetic status of this gene was evaluated on the basis of two microsatellites closed to *NRAS* locus (rs3220698 and rs3220987). PCR reactions were performed with fluorescent-labeled forward primers and the amplified PCR products were analyzed by capillary array electrophoresis on the ABI PRISM 3100 sequencer (Applied Biosystems, Foster City, USA) and GeneScan software (Applied Biosystems). Detection of loss of heterozygosity was performed as detailed in Loss of Heterozygosity Analysis Getting Started Guide. Two independent experiments were performed to confirm LOH results. The probability of one *NRAS* allele loss was evaluated as very high if only one allele was detected in each *NRAS* locus surrounding microsatellites (e.g. Fig. 1, case Y11.136). If only one of *NRAS* surrounding markers presented one microsatellite allele, this probability was evaluated as mean (e.g. Fig. 1, case Y14.711). The analysis was considered as inconclusive if both *NRAS* locus surrounding microsatellites gave non informative results (e.g. Fig. 1, case Y10.1471).

### FISH analysis

FISH analysis was performed on tissue microarray (TMA) containing 94 melanomas and on tissue sections from 7 melanoma patients. FISH probe preparation and FISH technique was performed as already described [20, 25]. All samples were analyzed with RP11-245I3 and RP11-269F19 probes covering *NRAS* and a region of chromosome 1 telomeric to *NRAS* gene (chr1:45.142.760–45.303.288, (2009 GRCh37/hg19)), respectively. Chromosome 1/*NRAS* disomie was concluded if two FISH signals for each probe were observed and polysomy if three or more FISH signals were detected in the majority of cell. Chromosome 1/*NRAS* signal ratios 2:1 and 1:2 were described as *NRAS* monosomy and *NRAS* gain, respectively. Innumerable *NRAS* FISH signals were interpreted as amplification. Intra-tumor heterogeneity was defined as a presence of cell populations with different chromosome 1/*NRAS* status; some cells with increase, some with normal and some with loss of *NRAS* allele.

### ATGC data analysis

Sequencing data about *NRAS* mutation of 479 cutaneous skin melanomas were extracted from cBioPortal platform as describe by Gao and colleagues [26]. The *NRAS* Q61 mutant allele percentage in 85 melanomas was compared to the data of our series.

### Statistical analysis

Overall survival (OS) was defined as the period between the date of the primary melanoma diagnosis to the date of death (all causes) or last follow-up evaluation. Survival was censored at the last follow-up evaluation. Distant metastasis free survival (DMFS) was defined as the period between the date of the primary melanoma diagnosis to the date of onset of stage IV melanoma or death. The date of onset of stage IV melanoma was defined as the date of the clinical examination or imaging procedure that provided an unequivocal diagnosis of distant visceral metastasis. Progression-free survival was defined as the period between the date of the primary melanoma diagnosis (with or without lymph node sentinel biopsy procedure) to the date of the first regional (node or cutaneous) recurrence (stage IIIB minimum). Progression-free survival, distant metastasis-free survival and overall survival curves were estimated using the Kaplan-Meier method, and differences between PFS, DMFS, and OS curves were assessed using the log-rank test.

Survival and histoprognostic markers (age, gender, Breslow index, ulceration and mitotic activity) of *NRAS* Q61 mutated primary melanomas were compared between two groups: one group of 48 melanomas with <60% of M%*NRAS* and another group of 24 tumors with ≥60% of M%*NRAS*.

Student tests were performed for quantitative values, and Chi^2^ tests for qualitative values. The results were considered significant when *P* < 0.05.

## Results

### *NRAS* mutation frequency and allele quantification

The frequencies of *BRAF* V600 and *NRAS* Q61 mutations were evaluated in 267 FFPE melanoma patients of the series (flow chart in Additional file 2: Figure S2) diagnosed between March 2013 and May 2015. *NRAS* mutation was detected in 48 melanomas, corresponding to 18% (48/267) of all melanomas and 33.8% (48/142) of *BRAF* V600 wild type cases. Additionally, 63 *NRAS* Q61 mutated melanomas, diagnosed before this period, were included into the molecular analyses. In total, 111 *NRAS* Q61 mutated tumors were collected with 60–95% tumor cells.

**Fig. 2:**
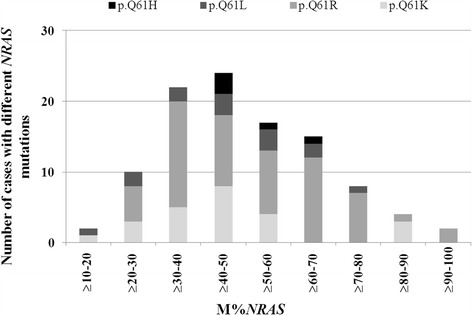
*NRAS* Q61 mutant allele burden in melanomas. Histogram representation of *NRAS* Q61 mutant allele quantity (in percentage) in 104 *NRAS* mutated melanomas. The X and Y axis correspond to the percentage of *NRAS* mutant and to the number of cases, respectively

Characterization of M%*NRAS* were performed in a larger group of 104 *NRAS* Q61 mutated melanomas containing ≥80% tumor cells. The corresponding mutations were c.182A > G p.Q61R in 58.6% (61/104), c.181C > A p.Q61K in 23.1% (24/104), c.182A > T p.Q61L in 13.5% (14/104), c.183A > T p.Q61H in 2.9% (3/104) and c.183A > C p.Q61H in 1.9% (2/104) of samples. M%*NRAS* was highly heterogeneous, ranging from 15.5 to 94% (Fig. 2). The majority of cases (60.6%, 63/104) had ≥30 to 60% M%*NRAS* and was thus considered as heterozygous (HET). The remaining 41 cases were considered as having non-heterozygous M%*NRAS*: 11.5% (12/104) had <30% of M%*NRAS* (Low non-HET) and 27.9% (29/104) had ≥60% of M%*NRAS* (High non-HET).

We then compared our results with database of the Cancer Genome Atlas (TCGA) analyzed on the CBioPortal platform. Among the 85 *NRAS* mutated cases that were available only 50.6% (43/85) had a heterozygous status of *NRAS* mutation, while 34.1% (29/85) was High non-HET and 15.3% (13/85) was Low non-HET (Additional file 3: Figure S3).

### FISH analysis

Among 101 samples (41 *NRAS* WT and 60 *NRAS* Q61) analyzed by FISH with *NRAS* locus and chromosome 1 specific fluorescent probes, different types of alternations were observed: no alteration of *NRAS*/chromosome 1 (disomy), disomy but rare cells with polysomy, polysomy and monosomy, which were detected in 23.1% (24/101), 19.2% (20/101), 9.6% (10/101) and 3.8% (4/101) of cases, respectively. *NRAS* amplification was a rare alteration and observed in 6.7% of melanoma samples (7/101). In 23.1% of samples (24/101), we observed intra-tumor cellular heterogeneity in *NRAS*/chromosome 1 copy numbers. Finally, FISH analysis of *NRAS* gene was non informative in 14.4% of cases (15/101).

**Fig. 3:**
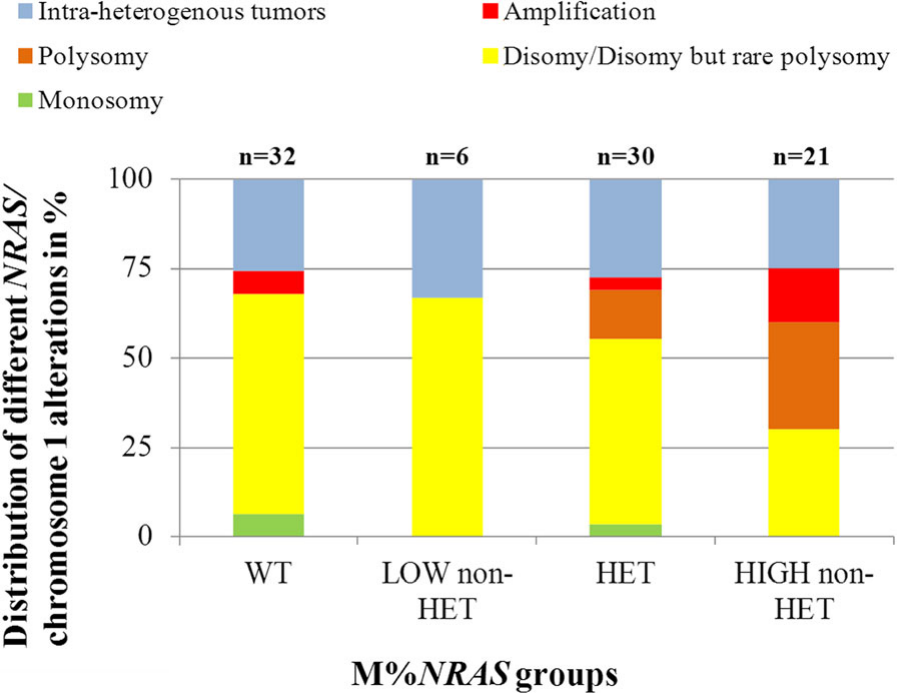
*NRAS*/chromosome 1 aberrations in *NRAS* mutated (*n* = 57) and *NRAS* WT (*n* = 32) melanomas. Histogram representation of prevalence of *NRAS*/chromosome 1 abnormalities evaluated by FISH in 104 *NRAS* mutated melanomas depending on the amounts of *NRAS* Q61 mutations. WT – wild-type, HET – heterozygous

To better understand the chromosomal mechanisms leading to M%*NRAS* increase, we compared M%*NRAS* and *NRAS*/chromosome 1 copy number status between 32 *BRAF*/*NRAS* WT and 57 *NRAS* Q61 mutated melanomas (Fig. 3). Disomy of *NRAS*/chromosome 1 (with or without polysomy in few cells) were detected in 59.4% (19/32) of *BRAF/NRAS* WT, 50% (15/30) of HET, but in only 28.6% (6/21) of High non-HET melanomas (*P* = 0.08). Polysomy of *NRAS*/chromosome 1 was detected in 13.3% (4/30) of HET, and 28.6% (6/21) of High non-HET samples, but was absent in *BRAF/NRAS* WT melanomas (*P* < 0.05). Amplification of *NRAS* was detected in 6.3% (2/32) and 9.5% (2/21) of *BRAF/NRAS* WT and High non-HET melanomas, respectively. Additionally, in 3.3% (1/30) of HET and 9.5% (2/21) of High non-HET cases, a gain of *NRAS* gene was observed. Deletion of *NRAS* was also a rare event and was observed in 9.4% (3/32) of *BRAF/NRAS* WT and 3.3% (1/30) of HET melanomas. A high intra-tumor copy number variation of *NRAS*/chromosome 1 was observed in 25% (8/32) of *BRAF/NRAS* WT, 30% (9/30) of HET and 23.8% (5/21) of High non-HET. FISH results of Low non-HET group were excluded from the comparative analysis because of insufficient numbers of samples (only 6 samples).

### Chromosome 1 microsatellite analysis

Eight polymorphic microsatellite markers were analyzed in 6 HET and 23 High non-HET tumors melanoma samples. In all HET melanomas, non LOH of *NRAS* locus surrounding microsatellites was detected but one for which microsatellites presented a low LOH, near the upper LOH detection threshold. In the majority of samples of High non-HET group, the microsatellite analysis revealed the presence of only one marker in the regions surrounding *NRAS* gene (Fig. 1). The probability of one *NRAS* allele loss was evaluated as very high in 30% (7/23) and mean in 26.1% (6/23) of tumors. In 26.1% (6/23) of tumors, the LOH results were non informative. In 17.4% (4/23), LOH with the presence of two alleles rather than one allele loss was detected.

### Correlation of M%*NRAS* and clinical data

**Fig. 4:**
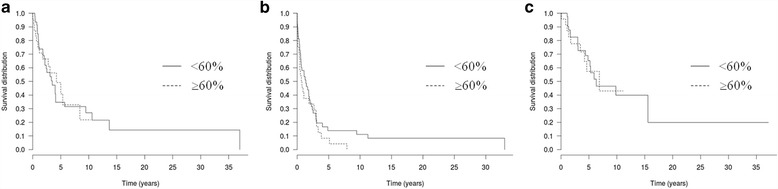
Survival curves. Kaplan-Meir survival curves of DMFS (**a**), PFS (**b**) and OS (**c**) of two distinct groups of *NRAS* mutated melanoma patients, according to the *NRAS* mutant allelic burden (<60% or ≥60%)

Information on primary tumor was available for 72 patients. The main histological subtypes were SSM and nodular melanomas, in 44.4% (32/72) and 38.9% (28/72) of cases, respectively. Median Breslow was 2.84 mm [0.4–10]. An ulceration was present in 47.2% (34/72) of melanomas, and 58.3% (42/72) had a mitotic activity (> 1 mitosis / mm^2^). Among these cases, we found *NRAS* Q61R mutation in 63.9% (46/72) of melanomas, *NRAS* Q61K in 25% (18/72), *NRAS* Q61L in 6.9% (5/72) and *NRAS* Q61H in 4.2% (3/72). M%*NRAS* was quantified by analysis of primary melanoma in 18.1% (13/72) of cases and of metastasis (lymph node, cutaneous or visceral) in 81.9% (59/72). Patients were divided into two groups depending on % of *NRAS* mutant allele. M%*NRAS* was ≥60% in the first group (*n* = 24, 33.3%) and <60% in the second cohort (*n* = 48, 66.7%). The second group contained 41 HET M%*NRAS* and 7 low not-HET M%*NRAS* cases. Clinical and pathological features of primary *NRAS* mutated melanomas were compared between these two groups and they are summarized in Table 2. No statistically significant differences were observed between these two groups with baseline criteria (*P* > 0.05). The median follow-up of the patients was 40 months (range [1–445]). PFS, DMFS and OS in both groups were not different (Fig. 4).

**Table 2:** Clinicopathologic characteristics of studied subjects. Comparison of clinical and pathological features of *NRAS* mutated primary melanomas according to the *NRAS* mutant allelic burden (<60% or ≥60%)

	≥60% οf *Μ*% *ΝRΑS n* = 24/72 (33.3%)	<60% οf *Μ*% *ΝRΑS n* = 48/72 (66.7%)	*P-value* (statistic test)
Origin of pyrosequencing sample			
From primary melanoma	5	8	0.91 (Chi2with Yates’ correction)
From metastasis	19	40	
Age			
Mean, years (SD)	63.1 ± 17.4	65.2 ± 14.7	0.61 (Student)
Median, year [range]	60 [37–93]	66 [32–97]	
Gender			
Ratio M/F	13/11	30/18	0.49 (Chi2)
Breslow index			
Mean, mm (SD)	3.0 ± 2.1	3.3 ± 2.2	0.57 (Student)
Median, mm [range]	3.00 [0.40–10.00]	2.61 [0.50–9.00]	
Ulceration	14	20	0.14 (Chi2)
Mitotic activity	11	31	0.45 (Chi2)
Initial AJCC stage			
I	5	10	0.7 (Chi2)
IA	4	2	
IB	1	8	
II	11	26	
IIA	4	10	
IIB	6	5	
IIC	1	11	
III	7	9	
IIIA	0	2	
IIIB	4	2	
IIIC	3	6	
IV	1	0	
NA	0	2	
Histologic subtype			
Nodular melanoma	9	19	0.63 (Chi2)
SSM	10	22	
Acral melanoma	1	0	
Lentigo maligna melanoma	0	1	
Mucosal melanoma	0	1	
On congenital naevus	0	1	
Inclassable	1	2	
NA	3	2	

*SMM* superficial spreading melanoma

*NA* data not available

*SD* standard deviation

*M/F* male/female

## Discussion

In this study, we reported the prevalence of *NRAS* Q61 mutation and, for the first time, the variations of *NRAS* mutant alleles (M%*NRAS*), in a large series of human melanoma samples. We have demonstrated that M%*NRAS* was highly heterogeneous; indeed, only 61% of *NRAS* mutated melanomas were heterozygous, while 30% of cases had a significantly increased M%*NRAS* (≥60%). Our results were confirmed by analysis of the cases of the TCGA database.

*NRAS* pyrosequencing assays used in this study were developed to identify all hot-spot mutations in the codon 61 of *NRAS* gene. The specificity of these assays for different mutations was confirmed by Sanger sequencing. Additionally, the genotyping accuracy of 40 *NRAS* mutated melanomas, 27 of which were p.Q61R was confirmed by immunohistochemistry with an antibody against Q61R [27]. In a recent study, we have demonstrated that pyrosequencing was a robust molecular technique for oncogenic mutant allele quantification, by comparing it with quantitative real time PCR and picodroplet digital PCR [20]. This previous study was focused on *BRAF* mutations, and similar M%*BRAF* heterogeneity was demonstrated in melanomas, with 19% of cases having an increased M%*BRAF*. Altogether, we estimate from both series that 36.2% of melanomas with *BRAF/NRAS* mutations have a non-heterozygous oncogenic allele.

Few studies have investigated M%*NRAS* in melanomas. Recently, we reported two cases with an increase of M%*NRAS* during metastatic melanoma progression; suggesting that M%*NRAS* may enhance metastatic capacities of melanomas [28]. Additionally, a large screening study of 833 cells lines from the database of Cancer Genome Project, Sanger Institute, focused on frequently mutated genes (six suppressor gene and five oncogenes), has identified *NRAS* homozygous mutation in 10% of cell lines [29]. However, the zygosity status was only determinate by manual examination of sequencing electropherograms.

Interestingly, in vitro studies of mutant RAS family members had demonstrated a high oncogenic potential of increased mutant allele frequency. The oncogenic potential of *NRAS* ^G12D/G12D^ was highly increased as compared to heterozygous or hemizygous *NRAS* cells in *NRAS*-driven hematopoietic transformation [30]. Additionally, progenitors of hematopoietic cells expressing the highest levels of *NRAS* ^G12D^ demonstrated cytokine-independent CFU-GM colony growth and exhibited an increased level of pAkt, pErk and pS6 proteins. Endogenous expression of *HRAS* ^G12V^ promotes papilloma and angiosarcoma development and these neoplasm initiations have been strongly associated with *HRAS* ^G12V^ allelic and gene copy number imbalances [31, 32].

Mutation in one allele of an oncogene is sufficient for activation of its targets and M%*NRAS* is expected to be around 50% in diploid cells. However, in tumours with high chromosome instability, chromosome number is rarely disomic and M%*NRAS* could widely exceeded 50%. To better understand the chromosome mechanisms leading to *NRAS* mutant allele increase in the proportion of *NRAS* mutated melanoma, we firstly performed FISH analyses with 2 BAC probes covering *NRAS* region and another region of chromosome 1, telomeric to this gene, in a large series of 104 melanomas. Different types of *NRAS*/chromosome 1 status were observed. Polysomy was mainly observed in *NRAS* mutated tumours and disomic and/or disomic but rare polysomic cells were less frequent in High non-HET M%*NRAS* than in M%*NRAS* WT tumours. Amplification and deletion of *NRAS* gene were rarely observed and were seen in both *NRAS* WT and *NRAS* mutated melanomas. Genomic analysis of human cutaneous melanoma genomes have been described in several studies. However, in most of them, only melanoma cell lines were studied. The analysis of 60 melanoma cell lines by Gast [33] have revealed targeted focal amplifications of *NRAS* genes in 11% of them (*n* = 7/60) and amplification were detected in both *NRAS* mutated and *NRAS* WT melanomas. This frequency is higher that the frequency of *NRAS* amplification detected in the present series and in other reports. In a subset of cutaneous melanocytic lesions, *NRAS* amplification was found to be restricted to a few cases with *NRAS* mutations [34]. Additionally, Stark and colleagues reported rare instances of focal amplification including *NRAS* gene in two cell lines with *NRAS* mutation; however, a poor correlation between copy number increase and concomitant mutation in this oncogene was described [35]. Polysomy of chromosome 1 and intra-tumour *NRAS*/chromosome 1 heterozygosity were frequently found in our series and was preferentially observed in *NRAS* mutated cancers. Correlation between mutant burden and gene copy gains have already been described for *KRAS* [36] and *BRAF* gene [20]. To our knowledge, *NRAS*/chromosome 1 copy number variations have never been described in melanomas with regard to the *NRAS* mutant allele burden.

Secondly, we analyzed chromosome 1 microsatellite polymorphism in normal and tumor DNA in a group of 29 *NRAS* mutated cancers by ALFP method. As expected, LOH with WT allele loss was mostly restricted to the High non-HET M%*NRAS* group. However, unlike in haematological malignancy [37, 38], acquired copy-neutral LOH was not a predominant mechanism of mutant allele imbalance in *NRAS* Q61 mutated melanomas; indeed this aberration was detected in only 23% of our samples. Other mechanisms of High non-HET M%*NRAS* were amplification and gain of *NRAS* gene (14%) and polysomy of chromosome 1 (23.8%). In 9 melanomas (38.1%), an intra-tumor copy number variation of *NRAS*/chromosome 1 was detected. As most melanomas have copy number variations of whole chromosomes and of chromosome segment, *NRAS* mutant allele increase could be a consequence of chromosome instability and clonality in these tumors.

Correlation of M%*NRAS* with clinical data revealed no association with age, sex, histological melanoma subtypes, nor with histoprognostic markers of the patients with *NRAS* Q61 mutated melanomas. Moreover, no differences in patient survival outcomes were observed between patients with <60% and ≥60% of M%*NRAS*. However, this cohort is a retrospective monocentric study, and the analyses were limited by small number of patients. Furthermore, the value of M%*NRAS* has to be investigated for prediction of response to targeted therapy, as done for M%*NRAS* with promising results. We hypothesize that High non-HET M%*NRAS* could have an oncogenic addiction effect, which could improve the sensitivity of targeted therapy in this subgroup of *NRAS* Q61 mutated melanoma.

## Conclusion

We report herein for the first time that 30% of cutaneous *NRAS* mutant melanomas have a high M%*NRAS*. Chromosome instability, (chromosome 1 polysomy, intra-tumor copy number variation of chromosome1/*NRAS*) rather than the acquired copy neutral LOH seems to be responsible for most of the cases with high M%*NRAS*. Histoprognostic markers and survivals were not different when comparing patients with <60% and ≥60% of M%*NRAS*; however this should be checked in a larger and multicentric series.

## Additional files

Additional file 1: Figure S1. Identification of *NRAS* WT and different *NRAS* mutations. Sequence of *NRAS* wild type allele (A) and of different *NRAS* mutant alleles and the corresponding pyrosequencing profiles. Pyrosequencing profiles by assays 1 (A, B, D, E) and by assay 2 (C and F) are present (TIFF 178 kb)

Additional file 2: Figure S2. Flow chart. Flow chart for melanoma samples regarding the *NRAS* gene status and the type of molecular analysis carried out (TIFF 170 kb)

Additional file 3: Figure S3. *NRAS* Q61 mutant allele burden in ATGC melanomas. Histogram representation of *NRAS* Q61 mutant allele quantity (in percentage) in 85 ATGC *NRAS* mutated melanomas. The X and Y axis correspond to the percentage of *NRAS* mutant and to the number of cases, respectively (TIFF 102 kb)

## Abbreviations

ALFP: Amplified fragment length polymorphism
DMFS: Distant metastasis free survival
FISH: Fluorescence in situ hybridization
HET: Heterozygous
LOH: Loss of heterozygosity
M%*NRAS*: *NRAS* mutant allele frequency
OS: Overall survival
PFS: Progression-free survival
