# Supplementary figures and images for: Variation of mutant allele frequency in NRAS Q61 mutated melanomas
Source: BMC Dermatol. 2017 Jul 1;17:9. doi: 10.1186/s12895-017-0061-x (PMC5494128; doi:10.1186/s12895-017-0061-x)

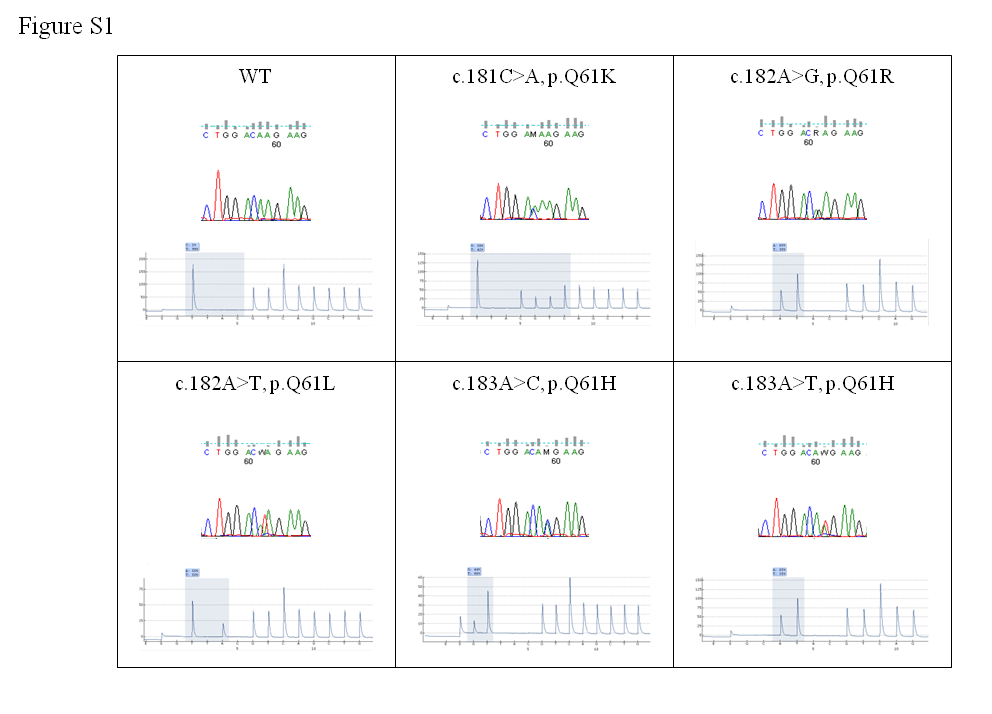

Supplement: Supplementary file 1 — Identification of NRAS WT and different NRAS mutations. Sequence of NRAS wild type allele (A) and of different NRAS mutant alleles and the corresponding pyrosequencing profiles. Pyrosequencing profiles by assays 1 (A, B, D, E) and by assay 2 (C and F) are present (TIFF 178 kb) [file 12895_2017_61_MOESM1_ESM.tif]

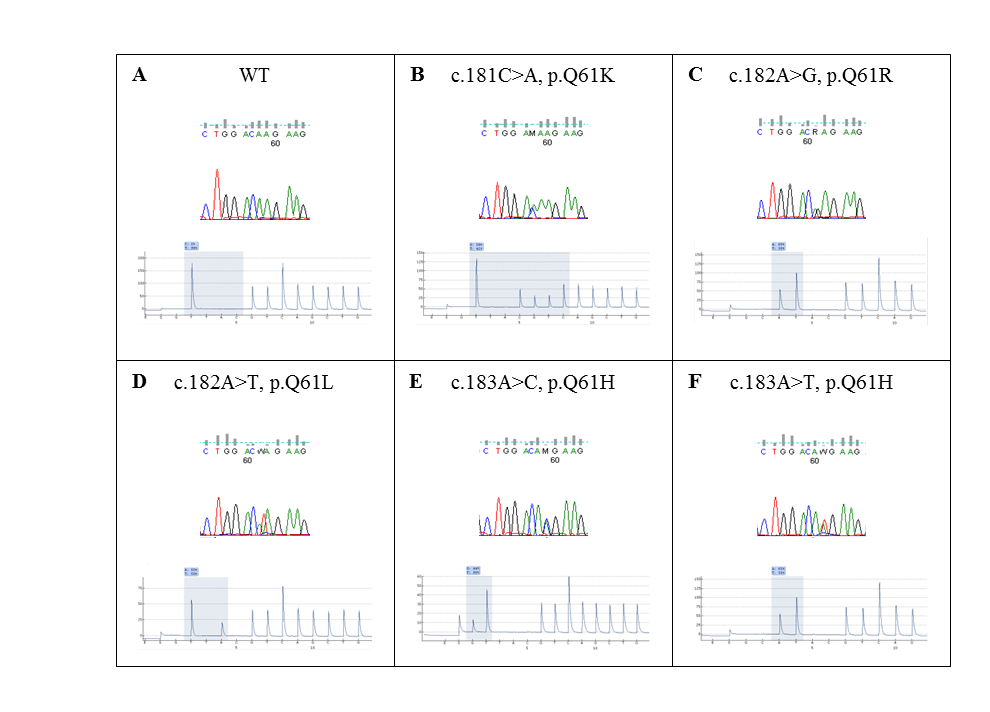

Supplement: Supplementary file 2 — Flow chart. Flow chart for melanoma samples regarding the NRAS gene status and the type of molecular analysis carried out (TIFF 170 kb) [file 12895_2017_61_MOESM2_ESM.tif]

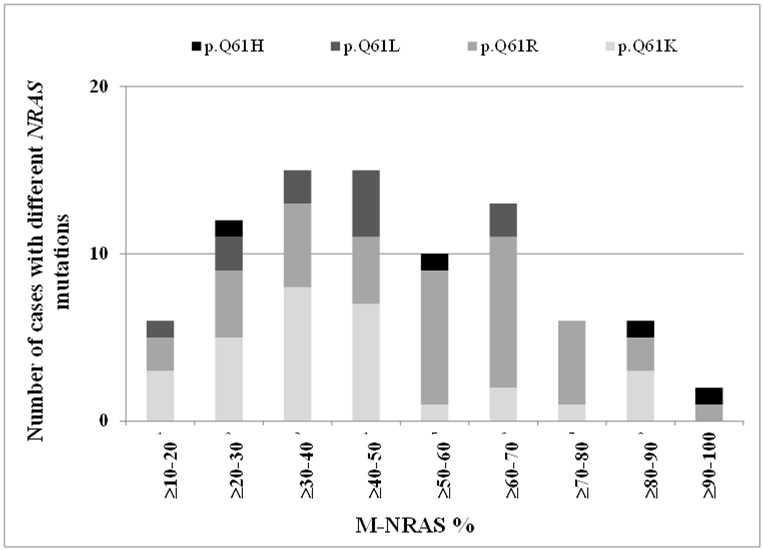

Supplement: Supplementary file 3 — NRAS Q61 mutant allele burden in ATGC melanomas. Histogram representation of NRAS Q61 mutant allele quantity (in percentage) in 85 ATGC NRAS mutated melanomas. The X and Y axis correspond to the percentage of NRAS mutant and to the number of cases, respectively (TIFF 102 kb) [file 12895_2017_61_MOESM3_ESM.tif]
